# Implementing Global Fund programs: a survey of opinions and experiences of the Principal Recipients across 69 countries

**DOI:** 10.1186/1744-8603-10-15

**Published:** 2014-03-24

**Authors:** Francis Wafula, Charles Marwa, David McCoy

**Affiliations:** 1 Aidspan, Nairobi, Kenya; 2 Queen Mary University, London, UK

## Abstract

**Background:**

Principal Recipients (PRs) receive money from the Global Fund to fight AIDS, Tuberculosis and Malaria (Global Fund) to manage and implement programs. However, little research has gone into understanding their opinions and experiences. This survey set out to describe these, thereby providing a baseline against which changes in PR opinions and experiences can be assessed as the recently introduced new funding model is rolled out.

**Methodology:**

An internet based questionnaire was administered to 315 PRs. A total of 115 responded from 69 countries in Africa, Asia, Eastern Europe and Latin America. The study was conducted between September and December 2012.

**Findings:**

Three quarters of PRs thought the progress update and disbursement request (PU/DR) system was a useful method of reporting grant progress. However, most felt that the grant negotiation processes were complicated, and that the grant rating system did not reflect performance.

While nearly all PRs were happy with the work being done by sub-Recipients (92%) and Fund Portfolio Managers (86%), fewer were happy with the Office of the Inspector General (OIG). Non-government PRs were generally less happy with the OIG’s work compared to government PRs.

Most PRs thought the Global Fund’s Voluntary Pooled Procurement system made procurement easier. However, only 29% said the system should be made compulsory.

When asked which aspects of the Global Fund’s operations needed improvement, most PRs said that the Fund should re-define and clarify the roles of different actors, minimize staff turnover at its Secretariat, and shorten the grant application and approval processes. All these are currently being addressed, either directly or indirectly, under a new funding model. Vigorous assessments should nonetheless follow the roll-out of the new model to ensure the areas that are most likely to affect PR performance realize sustained improvement.

**Conclusions:**

Opinions and experiences with the Global Fund were varied, with PRs having good communication with Fund Portfolio Managers and sub-Recipients, but being unhappy with the grant negotiation and grant rating systems. Recommendations included simplifying grant processes, finding performance assessment methods that look beyond numbers, and employing Local Fund Agents who understand public health aspects of programs.

## Introduction

Principal Recipients (PRs) implement programs for the Global Fund to fight AIDS, Tuberculosis and Malaria (hereafter referred to as ‘the Global Fund’ or ‘the Fund’) and/or recruit others to do so [1]. They are nominated by Country Coordinating Mechanisms (CCMs) and approved by the Fund following independent assessments by Local Fund Agents (LFAs) (Table 1) [2].

**Table 1:** Global Fund actors and their roles

**Global Fund Actor**	**Description of role**
**Fund Portfolio Manager (FPM)**	The Global Fund point person responsible for all communication between the Fund and the grant recipient country actors.
**Country Coordinating Mechanism (CCM)**	A country-level partnership that writes and submits grant proposals to the Global Fund based on defined country priorities.
Principal Recipient (PR)	Entities nominated or selected by CCMs to implement programs, or sub-contract other entities to implement the programs. The sub-contracted entities are referred to as ‘Sub-Recipients (SRs)’. Principal recipients may be government agencies, non-governmental organizations, private commercial firms or multilateral agencies.
Local Fund Agent (LFA)	Country-level organizations contracted by the Global Fund to oversee grant implementation on its behalf. Their roles include pre-grant assessment of country systems, and verification of information submitted by PRs.
Office of the Inspector General (OIG)	An independent unit of the Global Fund that ensures proper use of resources through audit, inspection and investigation of grants.

The ability of PRs to implement programs depends on their organizational capacity and their ability to work with other actors such as CCMs, LFAs and the Fund’s Secretariat. Although organizations need to demonstrate capacity before being selected as PRs [1,2], factors such as staff turnover and frequent changes in Global Fund requirements may limit their effectiveness. Their performance may also be affected by slow communication and poor relations with other actors, government bureaucratic bottlenecks and broader factors such as civil unrest.

The PRs’ importance in the Global Fund’s operations cannot be overemphasized. However, there are concerns over how well informed the Global Fund is on PRs, for instance, what constitutes an effective environment for sound implementation [3], and what support PRs need to improve their performance [4]. Partly in response to such concerns, the Global Fund recently adjusted its governance structure, allocating more staff towards grant management, and adopted a ‘New Funding Model’ [5]. However, the dearth of information on PRs makes it difficult to understand how these changes would affect opinions and experiences of PRs. The little that is known on PRs comes from country case studies [6-8]. There has been no survey of opinions and experiences across the broader PR group.

It is against this background that Aidspan developed the PR survey. The survey sought to describe the opinions and experiences of PRs on various aspects of the Fund operations. Aside from identifying areas most in need of improvement, the survey was designed to serve as a baseline against which PR opinions and experiences can be assessed as part of broader efforts to monitor and evaluate the effectiveness of the new funding model.

## Methodology

The study used a cross-sectional survey using a self-administered, internet-based questionnaire. The survey was conducted between September and December 2012.

A list of all PRs was obtained from the Global Fund website. Based on information retrieved in September 2012, there were 325 PRs operating in 139 countries from all eight Global Fund regions: namely: the East Asia and Pacific region, the Eastern Africa and Indian Ocean region, the Eastern Europe and Central Asia region, the Latin America and Caribbean region, the Middle East and North Africa region, the South and West Asia region, the Southern Africa region, and finally, the West and Central Africa region.

However, this typology creates groups that are too small for any meaningful analysis. For this reason, we classified PRs into two: government and non-government. The latter included private for-profit and not-for-profit organizations and multilateral organizations like the UN. Government and non-government PRs often take on different roles as PRs. The former, for instance, often leads treatment and prevention programs across the general population, while non-government PRs engage more with harder to reach groups such as the Most at Risk Populations (MARPS) and Persons with Disability (PWD). Additionally, the two types of PR organizations differ in structure and operations, meaning they are likely to encounter different types of challenges. For that reason, we considered this a useful classification for analysis.

We also classified PRs into two broad geographic regions for analysis; PRs from sub-Saharan Africa (SSA) and PRs from all the other regions (Table 2). We considered SSA a special category for two reasons. Firstly because the region has the highest burden of the three diseases [9], meaning SSA PRs are more likely to face a unique set of challenges, and secondly, because the region receives the largest share of money from the Global Fund [10], giving policy a strong incentive to understanding how their effectiveness can be maximized.

**Table 2:** The PR classification used for the survey

**PR type**	**Total number of PRs for each category (%)**
**Classification by PR type**	
Government PRs	164 (51%)
Non-government PRs	161 (49%)
**Classification by geographic region**	
Sub-Saharan Africa	128 (39%)
All other regions	197 (62%)

### Classification of PRs by type and geographic location

A short questionnaire was developed based on a literature review and Aidspan knowledge on PRs and the Global Fund more broadly. The instrument covered information on the nature and operations of PRs; experiences in grant implementation; relationships with other actors; and opinions on various Global Fund systems and processes. The tool also carried two open ended questions at the end: one seeking opinions on reasons for grant delays, and the other asking PRs what they think should be done to make the Global Fund more effective. The instrument was translated into French and Spanish, piloted on 10 PRs and adjusted accordingly, and a final internet-version developed by the Survey Monkey group (Survey Monkey®).

Email surveys have certain advantages over postal surveys, including lower costs and faster responses. However, they are also known to have lower response rates compared to interviewer administered questionnaires. A systematic review of response rates for the two types of surveys, for instance, found that internet surveys had an average response rate of 33%, compared to 56% for paper questionnaires [11,12]. We nonetheless opted for the email survey, as it was the most practical way of reaching recipients distributed across the globe.

The final tool was sent to all current PRs whose email addresses we had, with instructions that it be filled by persons most involved with the management of the organization’s programs. The email contacts were obtained from the Global Fund website. We sent a total of 315 emails; 156 and 159 to government and non-government PRs respectively. The emails explained that responses would be treated confidentially, and that identities of individual PRs and countries would not be disclosed. An incentive of an Amazon voucher worth $25 was included for each filled questionnaire. Two reminder emails were sent out, first after one week, and then after two and a half weeks. Responses were collated after a three-week waiting period.

Analysis was done using SPSS v20, with NVIVO 9 being used for the open-ended questions. The unit of analysis was the PR for all variables. Proportions were given for outcomes, including characteristics of organizations and opinions on various aspects of the Fund. Outcomes were reported, first across all PRs, then by PR type, and finally, by geographical region. Data were collected and analyzed across all disease components (rather than per disease), with the assumption that the disease component was unlikely to be a strong influence on PR opinions and experiences. Different questions had different response rates. We reported figures based on the number of responses obtained for each question.

### Findings

We received 115 completed questionnaires from 69 countries. Of these, 75 were in English, 22 in French and 18 in Spanish. Three-quarters of all responses came from non-government PRs (54% response rate, compared to a 19% response rate for government PRs).

There were fewer PRs from SSA (44%, compared to 56% for other regions). Nearly two-thirds had been PRs for over two years, with most having two or more Global Fund grants (Table 3).

**Table 3:** General characteristics of the PRs responded to the survey

**Characteristic (number of respondents)**	All PRs N* (%)	**Government N (%)**	**Non-government N (%)**
**Type of PR**	115	29 (25)	86 (75)
**Geographic location of PR (n=107)**			
Sub-Saharan Africa	47 (44)	13 (50)	33 (41)
Other regions	60 (56)	13 (50)	47 (59)
**Period as PR (n=113)**			
2 years and below	41 (36)	7 (23)	34 (41)
More than 2 years	72 (64)	22 (77)	49 (59)
**Total number of Global Fund grants (n=114)**			
One grant	49 (43)	7 (24)	42 (51)
Two or more grants	64 (57)	22 (76)	40 (49)
**Total annual expenditure of PR in USD (n=107)**			
10 million and less	57 (53)	16 (55)	41 (53)
11-30 million	23 (22)	6 (21)	17 (22)
Over 31 million	27 (25)	7 (24)	20 (25)

*-Some respondents did not respond to all questions, leading to variations in response rates for different questions.

#### Opinions on grant management and technical support

**Figure 1:**
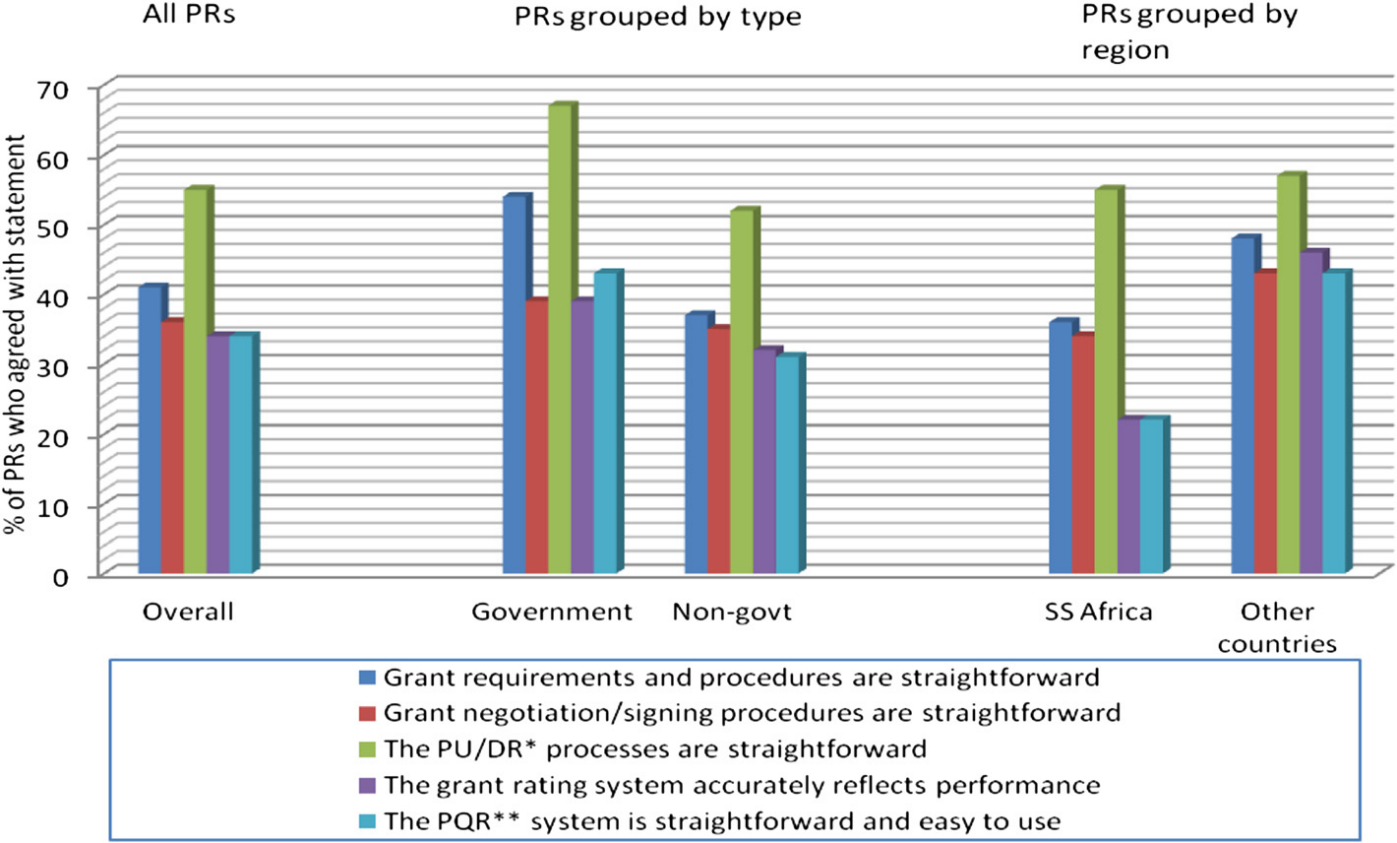
PR opinions on grant requirements and management.

**Figure 2:**
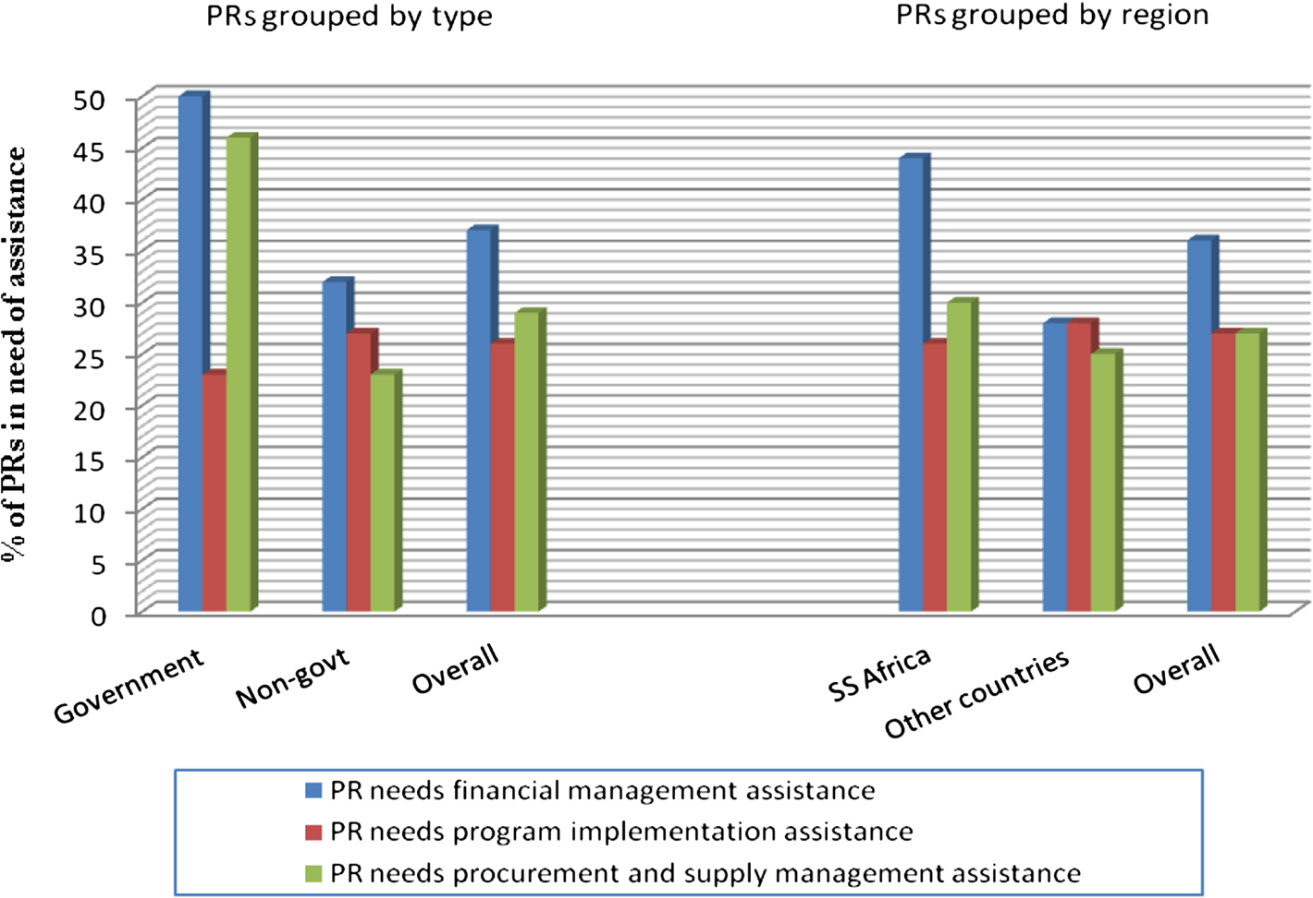
PR opinions on technical support requirements from the Global Fund.

Less than half of all PRs thought the grant negotiation processes were straightforward (Figure 1), with only one third saying they thought the Fund’s grant rating methodology reflected performance. More government PRs were happy with the grant management processes overall compared to non-government PRs. When asked whether they required technical support from the Fund in grant management, the majority, particularly non-government, felt they did not (Figure 2). PRs from SSA were more likely to say they required technical support.

**Figure 3:**
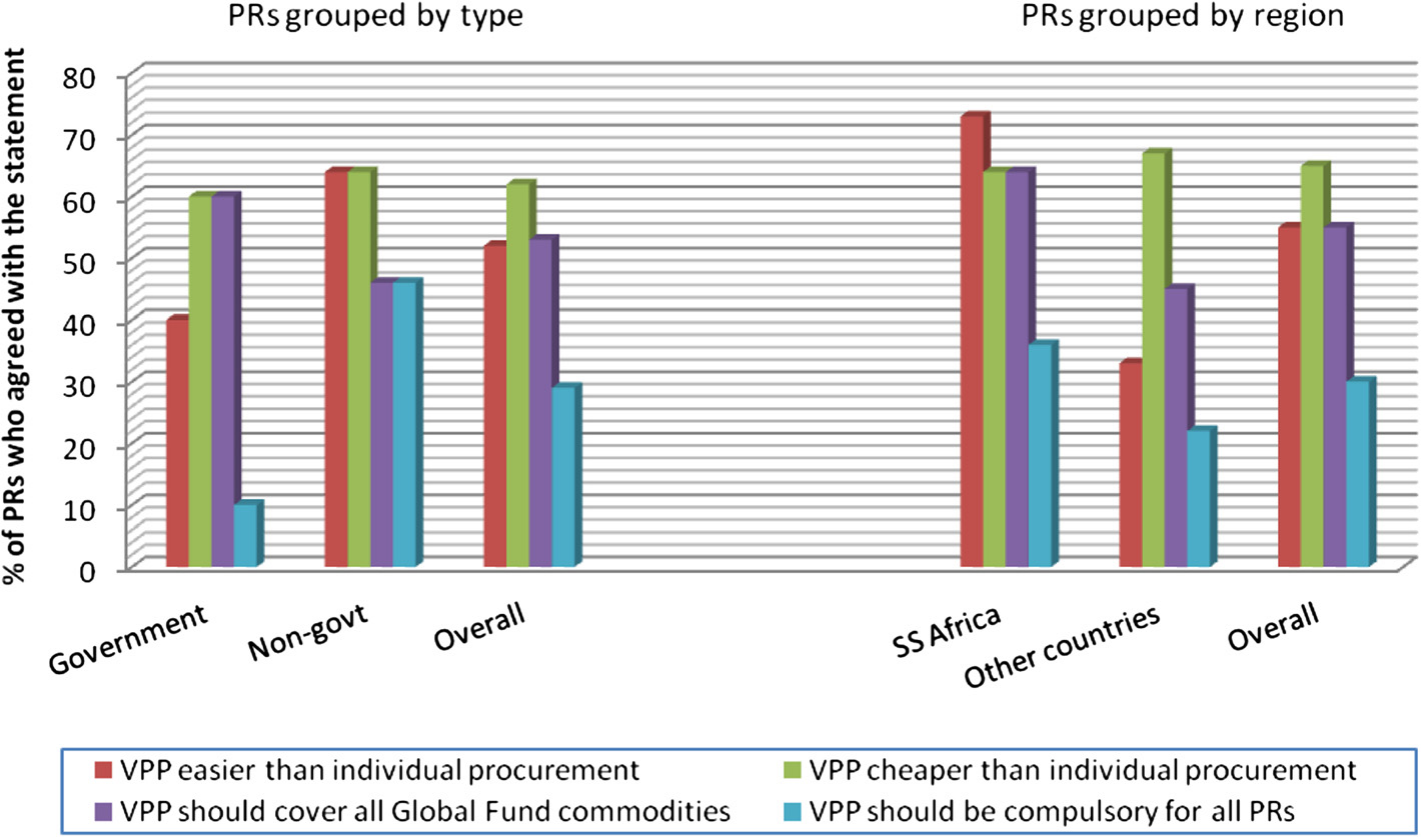
PR opinions on the voluntary pooled procurement system.

As far as procurement support was concerned, only 20% of PRs said their organizations had used the Fund’s Voluntary Pooled Procurement (VPP) system, with a proportionately higher number being government PRs. While nearly two thirds of those who had used the VPP system thought it made procurement cheaper, the suggestion to make it compulsory was objected overall (Figure 3).

#### Relationships of PRs with other global fund actors

Questions were asked on how PRs related with Fund Portfolio Manager (FPM), CCMs, LFAs and SRs. Government PRs were more likely to have a representative sitting on the CCM (68% compared to 43% for non-government). PRs from SSA were more likely to have a representative sitting in the CCM compared to other regions (57%, compared to 44%).

Over two-thirds of respondents thought SRs and FPMs responded to queries in good time (Table 4). However, only half were happy with the CCM response time, with non-government and SSA PRs being less happy overall.

**Table 4:** Principal Recipient communication and relationship with other Global Fund actors

	**Respondents who agreed with the statement**					
**Statement area**	**Overall number (%)**	**Government number (%)**	**Non-govt number (%)**	**Overall**	**SS Africa number (%)**	**Other countries number (%)**
**Adequacy of communication with other actors**						
Organization has adequate communication with FPM (*n=105*)	91 (86)	23 (82)	68 (88)	84 (87)	35 (80)	49 (92)
Organization has adequate communication with CCM (*n=105*)	76 (73)	24 (86)	52 (68)	72 (74)	29 (66)	43 (81)
Organization has adequate communication with LFA (*n=105*)	87 (82)	25 (89)	62 (81)	83 (86)	37 (84)	46 (87)
Organization has adequate communication with SRs (*n=91*)	84 (92)	21 (92)	63 (93)	81 (93)	35 (90)	46 (95)
**Response time to queries**						
FPM responds to queries in a timely manner (*n=104*)	74 (71)	15 (56)	59 (76)	69 (72)	25 (59)	44 (83)
CCM responds to queries in a timely manner (*n=105*)	54 (51)	17 (61)	37 (48)	50 (51)	18 (41)	32 (60)
LFA responds to queries in a timely manner (*n=105*)	61 (58)	17 (61)	44 (57)	59 (60)	20 (46)	39 (73)
SRs responds to queries in a timely manner (*n=91*)	61 (67)	13 (57)	48 (71)	60 (69)	21 (54)	39 (81)
**Overall nature of the working relationship**						
Organization has good relations with the FPM (*n=104*)	88 (84)	22 (82)	66 (86)	81 (84)	33 (77)	48 (90)
Organization has good relations with the CCM (*n=105*)	79 (75)	24 (86)	55 (71)	74 (76)	33 (75)	41 (77)
Organization has good relations with the LFA (*n=105*)	77 (74)	21 (75)	56 (73)	72 (74)	31 (80)	41 (77)
Organization has good relations with the SRs (*n=91*)	85 (93)	20 (87)	65 (96)	81 (93)	35 (90)	46 (95)

The nature of working relationships varied, with nearly all PRs saying they had good relations with SRs and FPMs (93 and 85% respectively) (Table 4). Government PRs were more likely to report good relations with CCMs (86% compared to 71% for non-government).

Over half of PRs had interacted with the Office of the Inspector General (OIG) in the past. Most felt relationships with the OIG were less cordial compared to other relationships. Only two-thirds, for instance, reported having good relations with the OIG, compared to 84% and 93% of PRs who said they related well with FPMs and SRs respectively. More government PRs were happy with the OIG compared to non-government PRs. Over 90% of the former said they thought the OIG was professional in its work, compared to only 49% for the latter.

Sub-Saharan Africa PRs had interacted more with the OIG, and were happier with its work compared to PRs from other regions. Nearly three-quarters of SSA PRs also said they were happy with the OIG’s work overall, compared to just over half of PRs from other regions.

#### Causes of delays in grant implementation

When asked about grant delays, over half the respondents (57%) said their organizations had experienced delays in grant implementation in the past. The delays were more common among government PRs (68% compared to 54% for non-government PRs).

“Disbursement delay” was the most commonly cited cause for delayed implementation. Reasons given for delayed disbursements included poor coordination between the PR and other actors, protracted grant negotiation processes, late submission of documents to the Fund’s Secretariat, and delays in receiving final approvals from the Secretariat.

PRs also mentioned inadequate support from LFAs and recipient country governments as causes for delay. LFAs were said to have inadequate expertise to oversee health programs. Governments were blamed for failing to facilitate programs, with PRs complaining of excessive bureaucracies, including delays in approving the use of donor funds in some places.

Lack of financial and programmatic management capacity were also linked to delays in program implementation. Some PRs, for instance, complained that SRs lacked capacity to collect and report financial data. High staff turnover and recruitment challenges were also identified as contributors to delays, particularly among PRs who provided services to stigmatized and criminalized groups.

Finally, some PRs blamed the Global Fund’s changing requirements for the delays. They complained that there were complexities involved in changing PR organizations’ systems and orienting staff whenever new grant management requirements were introduced.

#### PR suggestions on areas in need of improvement

When asked what aspects of the Global Fund were most in need of improvement, PRs identified three broad areas: 1) clarifying the functions of actors, 2) improving processes, and 3), building the capacity of PRs to adapt to new changes in the grant management system.

##### Clarifying the role and functions of actors

This was the most common suggestion. Most respondents asked that roles of CCMs and LFAs be better defined to reduce confusion and duplication. There were suggestions for the Fund to take a more proactive role in ensuring CCMs were properly constituted and regularly evaluated on performance.

*‘Our CCM is completely useless and the Global Fund doesn’t do a thing. There needs to be an independent review of performance with consequences for not fulfilling responsibilities. The CCM’s responsibilities seem to be divided and forced upon PRs’.* ***Non-government PR***

There were also concerns that a one-system-fits-all approach to governance was inappropriate, and that terms of reference for CCMs and LFAs should be made more adaptable.

Another suggestion was for the Global Fund to select LFAs that have good public health knowledge, as they would understand why certain programs were not implemented in ways that were originally proposed.

*‘An issue that should worry the Global Fund is the technical capacity of the LFA. Our LFA is good on financial issues, but it should strengthen its technical side at least in the three diseases and understand the reality of the health system’.* ***Government PR***

Aside from strengthening the role of in-country actors, there were suggestions to increase Global Fund presence in countries. Some PRs felt that the LFA was insufficient, and that the Fund should have an office where PRs can report issues and get quick responses. They also felt that this would enable the Fund to understand program implementation realities in the country.

*‘The Fund should consider a local staff (regional). Although we have very good communication with our portfolio manager, a local presence would give more attention and monitor country programs. The LFA functions as an accounting firm, and often does its job without assessing our comments…’* ***Non-government PR***

There are those who felt the Fund should also increase the number of grant management staff in Geneva to reduce waiting times and enable it to provide direct technical support to PRs. There were concerns over slow and poor communication of decisions from the Fund.

*‘Disbursement processing takes far too long without any explanation as to where we are in the process or when the transfer can be expected. It’s like you’re talking to the man behind the curtain in the Wizard of Oz sometimes.’* ***Non-government PR***

There were some concerns over what was termed as “excessively high turnover” of FPMs. Those who raised this concern felt that staff transfers slowed down program implementation, as new FPMs had to be given the same information as held by their predecessors.

##### Strengthening Global Fund processes and other areas of improvement

A number of PRs felt that the Fund should make grant negotiation processes easier for faster disbursements. There were suggestions to reduce the number of approval procedures, with some respondents expressing optimism that the new funding model would address this.

Concerns were expressed over confusion that followed periodic changes in the Fund’s requirements, with some PRs suggesting that a structured orientation system be introduced. This, they felt, would reduce the back and forth querying and speed up implementation.

There was also fear that the new funding model would render some countries ineligible for support. This was voiced by PRs from countries whose income classification had recently been revised upwards. The respondents recommended that the Fund specify clearly, which countries would be eligible, and what kinds of programs would be funded.

*‘We need clarity on continuity of funding after the current program cycle. Are middle income countries allowed to apply? And will there be specific MARPs (most at risk populations) streams under the funding?’* ***Non-government PR***

Another suggestion was that PRs be given more flexibility to re-program funds. They suggested the Fund develop guidelines on how money could be redirected to more urgent needs during implementation periods. This would strengthen the Fund’s commitment to the principle of country ownership and ensure that resources go towards high impact programs.

The Fund’s grant rating methodology was criticized for being overly numerical. Some PRs felt that a qualitative assessment component would give a better picture of performance, especially for programs that focused on health and community systems strengthening. Some also suggested that the Fund put more weight on contextual factors when interpreting performance.

*‘I think the Global Fund should appreciate a country’s operation environment. In our case we had an economic meltdown. This affected funding disbursements and delayed implementation, and as such, the country PRs lost their role to UNDP, yet they had the capacity to remain as PR. The performance was also affected during the transition period’.* ***Non-government PR***

## Discussion

Principal Recipients are responsible for the oversight and implementation of all Global Fund programs. For this reason, they represent an important intervention point, if meaningful improvements are to be achieved in the Fund’s overall performance. Few studies have described their opinions on what can be done to improve their work. The few studies we found were relatively old, focused mainly on understanding specific aspects of the PR, and were usually country specific [7,13-15]. Little attention was placed on PR opinions and experiences in the more recent literature [16,17]. This report presents findings from an internet-based survey of PRs from all eight Global Fund regions, the first independent survey of its kind.

The survey came at a time when the Global Fund was rolling out a new finding model, which seeks to increase the efficiency of the Fund, and promote value for money. For that reason, the study will provide a useful baseline for gauging how PRs opinions and experiences change with the new funding architecture.

The survey found that most PRs were somewhat unhappy with grant negotiation and signing processes, recommending that the processes be simplified, and that the Fund put more effort in explaining changes. The Fund has in the past been criticized for blaming PRs for poor grant performance without providing them with adequate guidance and support [4]. However, this is an area that the new funding model is designed to address, with the Fund introducing structures to support continuous dialogue between itself and recipients, and across in-country actors.

Another area of concern was re-programming of funds. On the one hand, some felt the Global Fund should provide clearer guidance on how money should be spent, while on the other hand, others thought PRs should have more discretion on how money is spent. The opposing views betray tension that is inherent in the Fund’s principles of country ownership (which gives countries discretion) and performance-based funding (which requires adherence to agreed targets). The Fund needs to balance between allowing re-programming within implementation periods and ensuring initially agreed targets are not altered excessively. This may become less of a problem with successful roll-out of the new funding model. The model is designed to, among other things, increase direct engagement between the Global Fund and the PRs in order to respond better to variations in country priorities and contexts [5].

Most PRs thought the grant rating methodology was not a fair reflection of performance. The Fund applies a standard grant performance assessment methodology, which guides decisions on subsequent disbursement amounts [18]. Grants are placed into one of five categories: A1 (exceeding expectations), A2 (meeting expectations), B1 (adequate), B2 (inadequate but potential demonstrated), and finally, C (unacceptable) [18]. Grants with a C rating will usually not receive subsequent disbursements [19]. As grant ratings are a central feature of the Fund’s performance-based financing, it is important that PRs are convinced that they reflect performance. However, some PRs felt that the current system had limited capacity to measure the more qualitative aspects of program performance. Similar views were expressed in an Aidspan analysis report on the Fund’s role in community systems strengthening [20]. Besides examining why some PRs have low confidence in the rating system, more effort should go towards exploring ways of integrating qualitative and quantitative measures of performance.

Reports that government bureaucracy and insufficient government support were slowing down grant implementation are worrisome. It is not the first time PRs have raised this concern. In Tanzania, for instance, the requirement that all foreign aid go through the finance ministry was linked with delays in program implementation. In Ethiopia, excessive government bureaucracies were linked to massive delays in procurement of insecticide-treated nets [6,21]. Governments should do more to reduce these bottlenecks and speed up implementation.

Most PRs felt they did not require direct assistance from the Global Fund. However, there were calls to develop systems that will ensure recipient organisations are well informed about changes in the Fund’s requirements and procedures. Past studies have linked PR capacity problems to high staff turnover and changing requirements from the Global Fund [6,22]. Some respondents complained that high FPM turnover slowed down their work. Different FPMs have in the past been reported to have different demands from in-country actors, causing confusion and slowing down program implementation [23]. This could be reduced if country communications were channeled to teams rather than individuals at the Fund, and if Global Fund requirements were standardized and made sufficiently clear. The Fund has in recent times established country teams to enhance collaboration across different clusters at the Fund, and improve and harmonize grant management decisions [24].

The majority of PRs who had used the VPP system thought it made procurement easier. This is in line with the Fund’s own assessment, which linked the VPP to better commodity governance, lowered prices, improved terms and conditions from suppliers [25,26].

While the VPP’s value was acknowledged, the suggestion to make it compulsory was opposed, particularly among government PRs. A number of reasons may explain this. It may be that government agencies have sufficient capacity and experience in procurement, or it may reflect a pursuit of self interest among staff which is easier under a non-compulsory procurement system. Procurement of drugs has been widely linked to corruption; observers have estimated in the past that 10–25% of public procurement resources are lost to corruption in poor countries [27,28]. Although the VPP is voluntary, the Secretariat may require a PR to use it if they have inadequate procurement capacity [29,30]. It is important that underlying reasons for resisting the suggestion to make it compulsory be explored in future surveys.

Nearly all PRs thought their communication and working relations with the FPMs and SRs were good (which we call vertical communication). However, fewer felt the same about CCMs and LFA, the other in-country actors (we call this horizontal communication). Poor horizontal communication has been documented in the past, with CCMs being blamed for failing to provide leadership [15]. A previous evaluation found that only half of CCMs had documented ways of conducting PR oversight activities [31]. While CCMs are an innovative governance concept, their success depends on effective communication with other in-country actors.

One reason why CCMs may not carry out oversight roles effectively is the presence of PR staff on the CCM. This was reported mainly among government PRs and in SSA. Having PR staff on the CCM creates conflict of interest. The CCMs have to nominate PRs through a transparent process. However, experience shows that this may not happen where conflict of interest exists. The decision to pick a PR in Uganda, for instance, was reportedly influenced by the CCM chair, who had an affiliation with the organization that was selected [32].

While the presence of PR staff creates conflict of interest, requiring that they be excluded is not without problems. In Zambia, for instance, removal of PR staff from the CCM resulted in reduced CCM oversight activity [6]. This calls for more innovative thinking around CCMs’ composition, including the possibilities of having alternate CCM membership, or requiring that members with conflict of interest abstain from voting on certain issues. However, countries need to take the lead in minimizing CCM conflict of interest because direct involvement by the Fund may be perceived as going against the principle of country ownership.

Communication with LFAs was also problematic, with PRs saying the LFAs lacked an understanding on the health system. Similar views have been expressed elsewhere. Past assessments have shown LFAs to have good financial management skills, but limited knowledge on health-related issues [14,23,33,34]. One of the recommendations from a survey in Uganda, for instance, was that the country should form stronger relationships between the Global Fund actors and technical country-based partners whose health sector capacity was higher than that of LFAs [32].

To date, LFAs have operated as complete packages, offering financial and programmatic oversight activities on behalf of the Fund. Going forward, the Fund should put more effort in assessing the capacity of LFAs to deliver on both fronts; where inadequacies are observed, the LFA should be compelled to strengthen their capacity before assuming LFA functions.

It is not clear why the government and non-government PRs had such varied views on the OIG. While over half of non-government PRs thought the OIG’s conduct was not professional, nearly all government respondents thought the inspector’s office conducted its activities professionally. Similarly, more SSA PRs thought the OIG was doing its job professionally. While these may be genuine differences in opinions on the OIG, it may also reflect a bias, where government and SSA PRs did not want to appear as painting the OIG in bad light. The latter is a real possibility, considering that governments and SS-African countries are the largest beneficiaries of the Global Fund.

The OIG has been instrumental in minimizing grant governance problems and financial mismanagement, leading to improved used of funds [35]. However, the office increasingly received criticism for the manner in which it operated, leading to the dismissal of the head in November 2012 [36]. A new Inspector General has since been appointed. Future research should aim to understand factors that influence how the OIG interacts with PRs, and to examine whether the difference between the different PR categories are genuine.

There were some limitations in the survey. Although the survey was sent to nearly the same number of government and non-government PRs, the majority of responses came from the latter group. This may reflect a lack of accuracy in our email contacts list for government PRs, or it may be an indication of a higher willingness to respond among non-government PRs.

It may also be that the emails landed on the “wrong” desk, something that is more likely to happen in government organizations that would normally have a higher number of staff and departments. Future research should explore reasons for the response rate variations, and examine whether this reflects broader communication challenges or problems between the various Global Fund actors (for instance, whether government PRs respond slower/poorly to queries from the FPMs or LFAs). Regional variations in response rates were minimal overall.

Many respondents also skipped a question or two, presumably because they felt they were not well placed to answer them. While care was taken to ensure the questions were broad enough to be answered by one person with good knowledge of the organization, it is possible that respondents did not have certain information at hand, or did not trust that the information they had was accurate. Another probable reason for skipping questions is respondents fearing to paint their organizations as inadequate or lacking capacity to undertake certain roles.

Finally, although the survey was administered in English, French and Spanish, there is a real chance that respondents who do not speak any of the three languages would have failed to respond. A number of Global Fund supported countries speak other languages, including Russian, Portuguese, Arabic and many others.

## Conclusion

We sought to understand grant application and implementation experiences of PRs, and get opinions on which aspects of the Global Fund operations need improvement.

Opinions and experiences with the Global Fund were varied, with PRs having good communication with FPMs and SRs, but being unhappy with the grant negotiation and grant rating systems. Recommendations included simplifying grant processes, finding performance assessment methods that are not limited to measuring numbers, and exploring why PRs were unhappy with the OIG. The Global Fund should also minimize the turnover of its staff in order to reduce grant implementation delays.

## Competing interests

The authors declare that they have no competing interests.

## Authors’ contributions

FW designed the survey, collected the data, performed the analysis, interpreted the data, and drafted the manuscript. CM helped design the questionnaire, and was involved in data collection and analysis. DM participated in formulating the study, developing the questionnaire, and drafting the manuscript. All authors read and approved the final manuscript.

## References

[B1] Aidspan A Beginner’s Guide to the Global Fund 2011 2 Nairobi, Kenya: Aidspan http://www.aidspan.org

[B2] GFATM LFA Guidelines for the Principal Recipient (PR) Assessment Global Fund to Fight AIDS 2007 Geneva, Switzerland: Tuberculosis and Malaria

[B3] Lu C Michaud CM Khan K Murray CJ Absorptive capacity and disbursements by the Global Fund to Fight AIDS, Tuberculosis and Malaria: analysis of grant implementation Lancet 2006 368 9534 483 488 10.1016/S0140-6736(06)69156-3 16890835

[B4] Hruska AJ The Global Fund’s principal recipients . . . or neglected partners Lancet 2004 364 918 10.1016/S0140-6736(04)17039-6 15364175

[B5] GFATM The New Funding Model: Key features and implementation Version 6.2, December 2012 2012 Geneva: Global Fund to Fight AIDS, Tuberculosis and Malaria

[B6] Brugha R donoghue M Starling M Walt G Ssengooba F Pariyo G Cliff J Fernandes B Nhatave I Ndubani P Mwale S Global Fund Tracking Study: Country Summaries and Conclusions 2005 LSHTM, University of Eduardo Mondlane, Ministry of Health- Maputo, Makerere University, University of Zambia

[B7] Mtonya B Chizimbi S Systemwide Effects of the Global Fund in Malawi: Final Report 2006 Bethesda, Maryland: Abt Associates

[B8] Starling M Brugha R Walt G Cliff J Fernandes B Global Fund Tracking Study: Mozambique Country Report 2005 LSHTM, University of Eduardo Mondlane, Ministry of Health- Maputo

[B9] Vitoria M Granich R Gilks CF Gunneberg C Hosseini M Were W Raviglione M De Cock KM The global fight against HIV/AIDS, tuberculosis, and malaria: current status and future perspectives Am J Clin Pathol 2009 131 6 844 848 10.1309/AJCP5XHDB1PNAEYT 19461091

[B10] Avdeeva O Lazarus JV Aziz MA Atun R The Global Fund’s resource allocation decisions for HIV programmes: addressing those in need J Int AIDS Soc 2011 14 51 10.1186/1758-2652-14-51 22029667 PMC3223126

[B11] Nulty D The adequacy of response rates to online and paper surveys: what can be done? Assessment Eval High Educ 2008 33 3 301 314 10.1080/02602930701293231

[B12] Ogier J The response rates for online surveys - a hit and miss affair. Paper presented at the 2005 Australian Evaluations Forum: University Learning and Teaching: Evaluating and Enhancing the Experience 2005 Sydney: UNSW

[B13] Biesma R Makoa E Mpemi R Tsekoa L Odonkor P Brugha R The implementation of a global fund grant in Lesotho: applying a framework on knowledge absorptive capacity Soc Sci Med 2012 74 3 381 389 10.1016/j.socscimed.2011.07.020 21907474

[B14] Brugha R donoghue M Starling M Walt G Ssengooba F Pariyo G Cliff J Fernandes B Nhatave I Ndubani P Mwale S Global Fund Tracking Study: A Cross-Country Comparative Analysis 2005 LSHTM, University of Eduardo Mondlane, Ministry of Health- Maputo, Makerere University, University of Zambia

[B15] Kapiriri L Martin DK The Global Fund Secretariat’s suspension of funding to Uganda: how could this have been avoided? Bull World Health Organ 2006 84 7 576 580 10.2471/BLT.06.030718 16878232 PMC2627384

[B16] Samb B Evans T Dybul M Atun R Moatti JP Nishtar S Wright A Celletti F Hsu J Kim JY Brugha R Russell A Etienne C World Health Organization Maximizing Positive Synergies Collaborative G An assessment of interactions between global health initiatives and country health systems Lancet 2009 373 9681 2137 2169 19541040 10.1016/S0140-6736(09)60919-3

[B17] Sherry J Mookherji S Ryan L The Five-Year Evaluation of the Global Fund to Fight AIDS, Tuberculosis, and Malaria: Synthesis of Study Areas 1, 2 and 3 The George Washington University School of Public Health and Health Services, The Johns Hopkins Bloomberg School of Public Health 2009 Macro International Inc

[B18] GFATM Performance Based Financing website Geneva, Switzerland http://www.theglobalfund.org/en/performancebasedfunding/decisionmaking/methodology/ (accessed July 2012)

[B19] GFATM PR Performance Analysis by Sector for all Grants from 2005 to 2010 2011 Geneva, Switzerland: GFATM

[B20] Decosas J McCoy D The Global Fund and Community Systems Strengthening: The Wrong Organisation for the Right Job? Or the Right Organisation Doing the Job Wrongly? Report prepared for Aidspan 2012 Nairobi: Aidspan

[B21] Nahlen BL Low-Beer D Building to collective impact: the Global Fund support for measuring reduction in the burden of malaria Am J Trop Med Hyg 2007 77 6 Suppl 321 327 18165509

[B22] Brugha R Donoghue M Starling M Ndubani P Ssengooba F Fernandes B Walt G The Global Fund: managing great expectations Lancet 2004 364 9428 95 100 10.1016/S0140-6736(04)16595-1 15234862

[B23] GAO Global Health: Global Fund to Fight AIDS, TB and Malaria Has Improved Its Documentation of Funding Decisions but Needs Standardized Oversight Expectations and Assessments. Report to Congressional Committees 2007 Washington D.C: Government Accountability Office report number GAO-07-627

[B24] GFATM LFA Manual 2011 Geneva, Switzerland: Global Fund to fight AIDS, Tuberculosis and Malaria

[B25] GFATM Procurement Support Services Progress Report: June 2009 – Dec 2010: Supporting Grant Implementation & Influencing Market Dynamics for HIV/AIDS and Malaria Products 2011 Geneva, Switzerland: GFATM

[B26] GFATM Voluntary Pooled Procurement: Key Results (2009 - 2011) 2012 Geneva, Switzerland: Global Fund to Fight AIDS, TB and Malaria

[B27] Transparency International Handbook for Curbing Corruption in Public Procurement 2006 Jakarta, Indonesia: Transparency International

[B28] WHO Medicines: Corruption and Pharmaceuticals, Fact Sheet No. 335 2009 Geneva: World Health Organization

[B29] GFATM The Global Fund: Fifteenth Board Meeting, Geneva, Switzerland, 25-27 April 2007 2007 Geneva, Switzerland: GFATM

[B30] GFATM The Global Fund: Report of the Market Dynamics and Commodities Ad hoc Committee (Twenty-First Board Meeting, Geneva, Switzerland, 28-30 April 2010) 2010 Geneva, Switzerland: GFATM

[B31] GFATM Report on the Assessment of Country Coordinating Mechanisms: Performance Baseline 2005 Geneva, Switzerland: Technical Evaluation Reference Group, Global Fund to Fight AIDS, TB and Malaria

[B32] Donoghue M Brugha R Walt G Pariyo G Ssengooba F Global Fund Tracking Study – Uganda country report 2005 Uganda: London School of Hygiene & Tropical Medicine, UK, and Institute of Public Health, Makerere University

[B33] Benzason K Replenishing the Global Fund: An Independent Assessment 2005 Geneva, Switzerland: Report Commissioned by the Vice-Chair of the Replenishment as an Independent Assessment

[B34] Euro Health Group Evaluation of the Local Fund Agent System, Final Report prepared for the Global Fund to Fight AIDS, TB and Malaria 2007 Soborg, Denmark: Euro Health Group

[B35] Aidspan OIG Audits lead to actions to strengthen CCMs in Chad and Kazakhstan Glob Fund Obs 2012 207

[B36] Aidspan Global fund fires inspector general Glob Fund Obs 2012 202

